# Supplementary material for: Engineering surface atomic structure of single-crystal cobalt (II) oxide nanorods for superior electrocatalysis
Source: Nat Commun. 2016 Sep 21;7:12876. doi: 10.1038/ncomms12876 (PMC5035995; doi:10.1038/ncomms12876)
Supplement: Supplementary Information — Supplementary Figures 1-23, Supplementary Tables 1-7, Supplementary Notes 1-5, Supplementary Methods and Supplementary References [file ncomms12876-s1.pdf]

Supplementary Figures

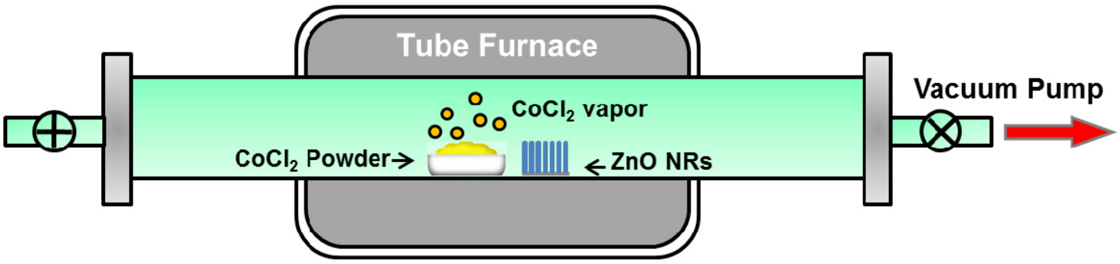

Supplementary Figure 1. Experimental setup illustrating the cation exchange process in gas phase.

The carbon fiber paper (CFP) substrate loaded with ZnO nanorods (NRs) is placed in the center of the furnace tube and $CoCl_2$ powder is placed upstream from the center of the tube. A complete cation exchange is accomplished by heating the tube at 600 °C for 30 min in Ar gas flow.

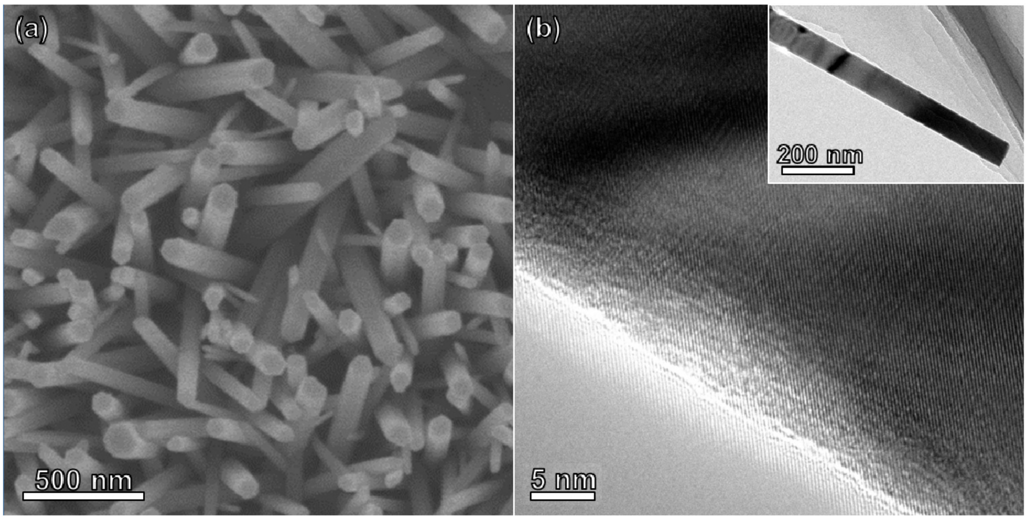

Supplementary Figure 2. (**a**) Top-view scanning electron microscopy (SEM) image of ZnO NRs. (**b**) High resolution transmission electron microscopy (HRTEM) image of a single ZnO NR, with the low magnification TEM image displayed in the inset, showing the smooth surface of the initial ZnO template.

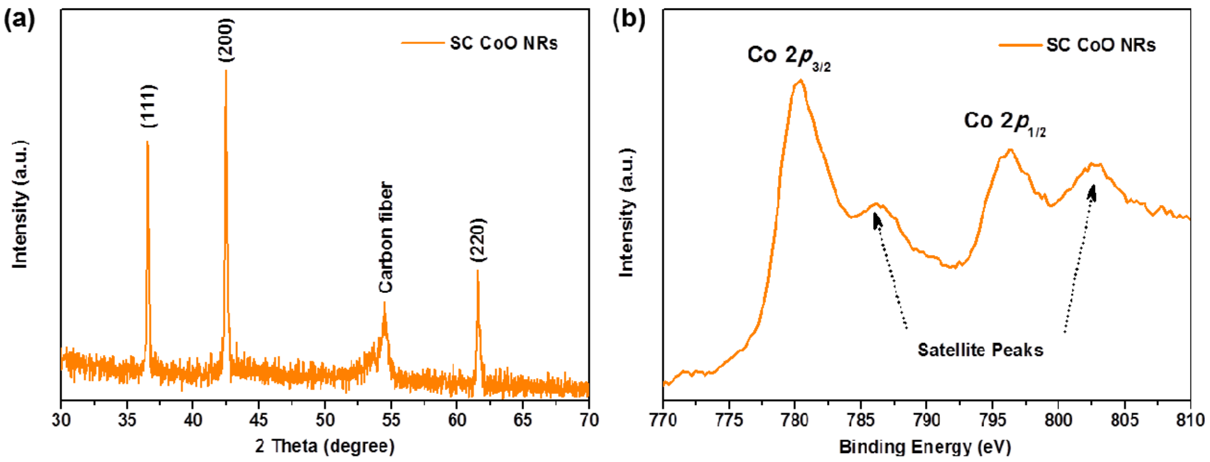

Supplementary Figure 3. (**a**) X-ray diffraction (XRD) and (**b**) Co 2*p* X-ray photoelectron spectroscopy (XPS) spectra of single-crystal (SC) CoO NRs, confirming the successful fabrication of CoO NRs by the cation exchange process in gas phase. The satellite peaks indicated by the arrows in (**b**) confirm the existence of Co^{2+}.

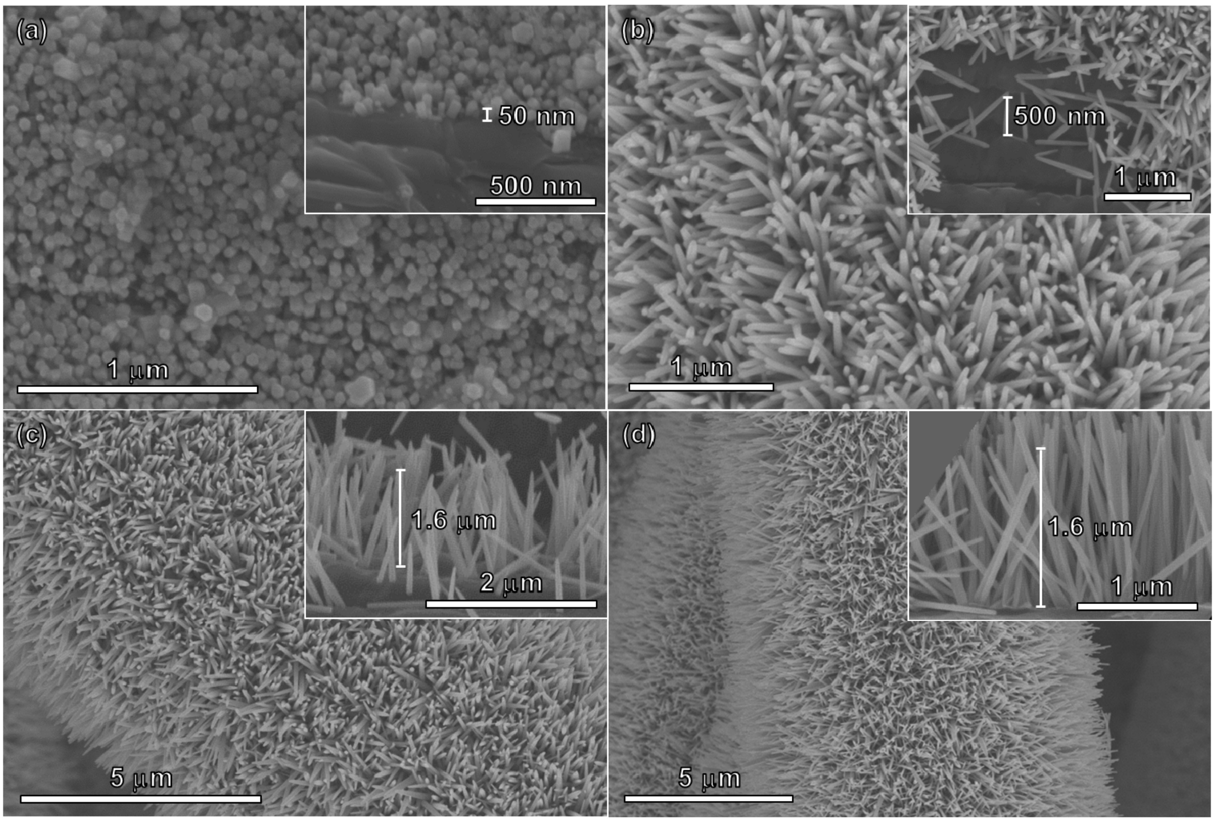

Supplementary Figure 4. (**a**)-(**d**) Top-view SEM images of ZnO NRs grown for 1, 2, 3 and 6 h, respectively. The insets in (**a**)-(**d**) are the side-view SEM images of ZnO NRs showing that the lengths of NRs grown for 1, 2, 3 and 6 h are approximately 50 nm, 500 nm, 1.6 μm and 1.6 μm, respectively.

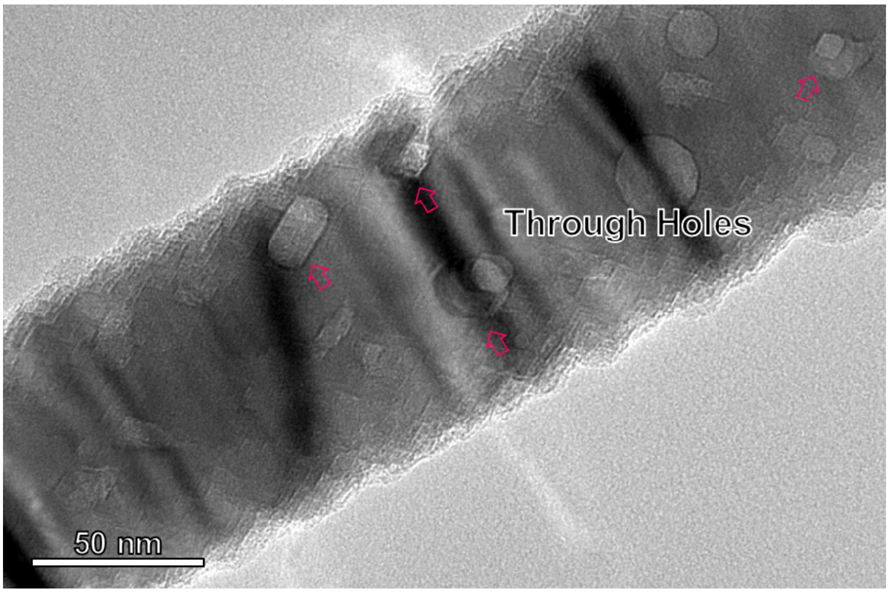

Supplementary Figure 5. High-magnification TEM image of an individual SC CoO NR exhibiting the through holes across the NR.

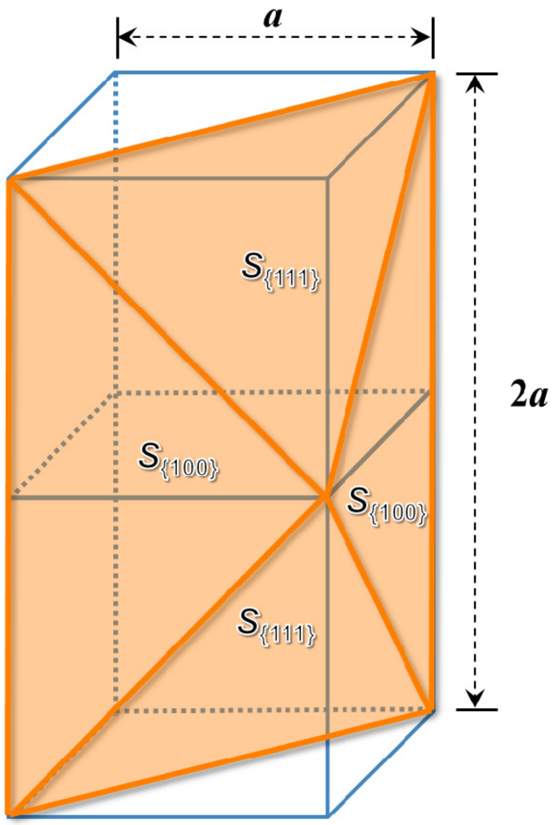

Supplementary Figure 6. Illustration of an individual nanopyramid in cubic lattice.

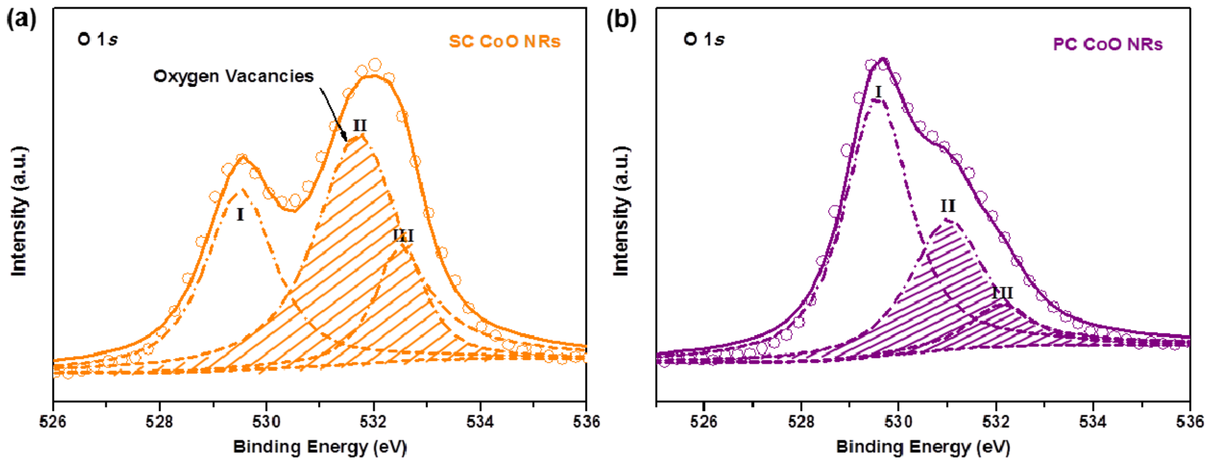

Supplementary Figure 7. XPS O 1*s* spectra of (**a**) SC and (**b**) PC CoO NRs. The circles and lines are experimental and deconvolution data, respectively. The peak with low binding energy (Peak I) centered at 529.5 eV is attributed to the lattice oxygen (O_L)[1], whereas peak II with middle binding energy (531.0-531.5 eV) is assigned to a large number of defect sites with lower oxygen coordination[2,3], and peak III with high binding energy (532.0-532.4 eV) is associated with hydroxyl species of the surface-adsorbed water molecules[3]. Obviously, the area of peak II of SC CoO NRs is much higher than that of polycrystalline (PC) CoO NRs, indicating that a larger number of O-vacancies is present on the surface of SC CoO NRs.

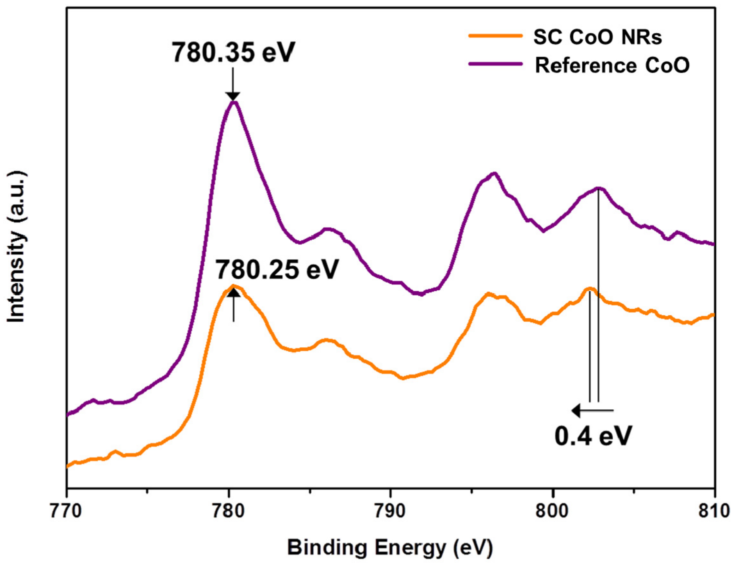

Supplementary Figure 8. Co 2*p* XPS spectra of SC CoO NRs and commercial CoO powder (reference CoO).

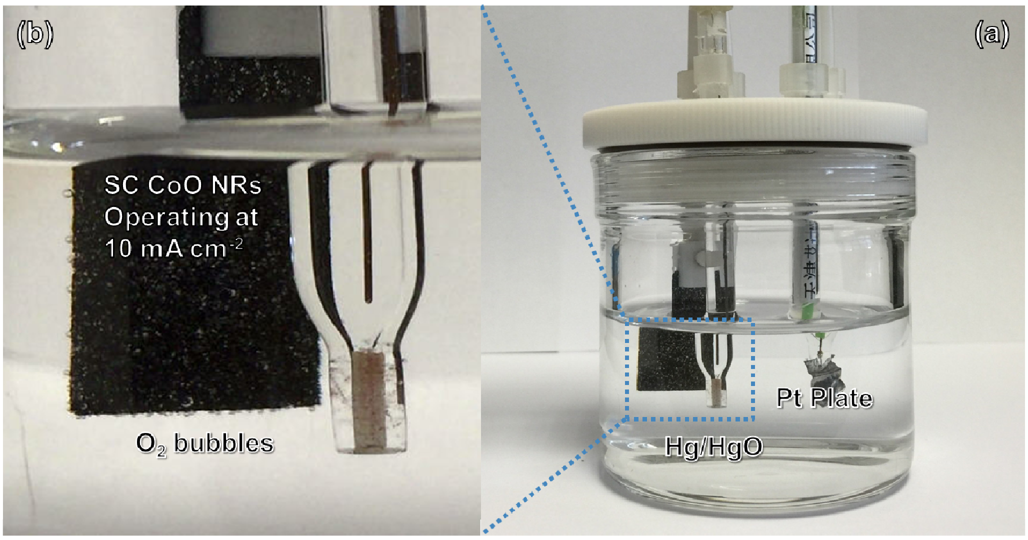

Supplementary Figure 9. (a) Photograph of a home-made electrochemical cell for the oxygen reduction reaction (ORR) and oxygen evolution reaction (OER) measurements. The three electrode system consists of an Hg/HgO reference electrode, a Pt plate as the counter electrode and the synthesized catalyst on CFP as the working electrode. (**b**) The enlarged photograph of SC CoO NRs loaded on CFP operating at 10 mA cm^{-2} in OER. Bubbles are generated on the surface of CFP indicating a continuous formation of O_2 gas.

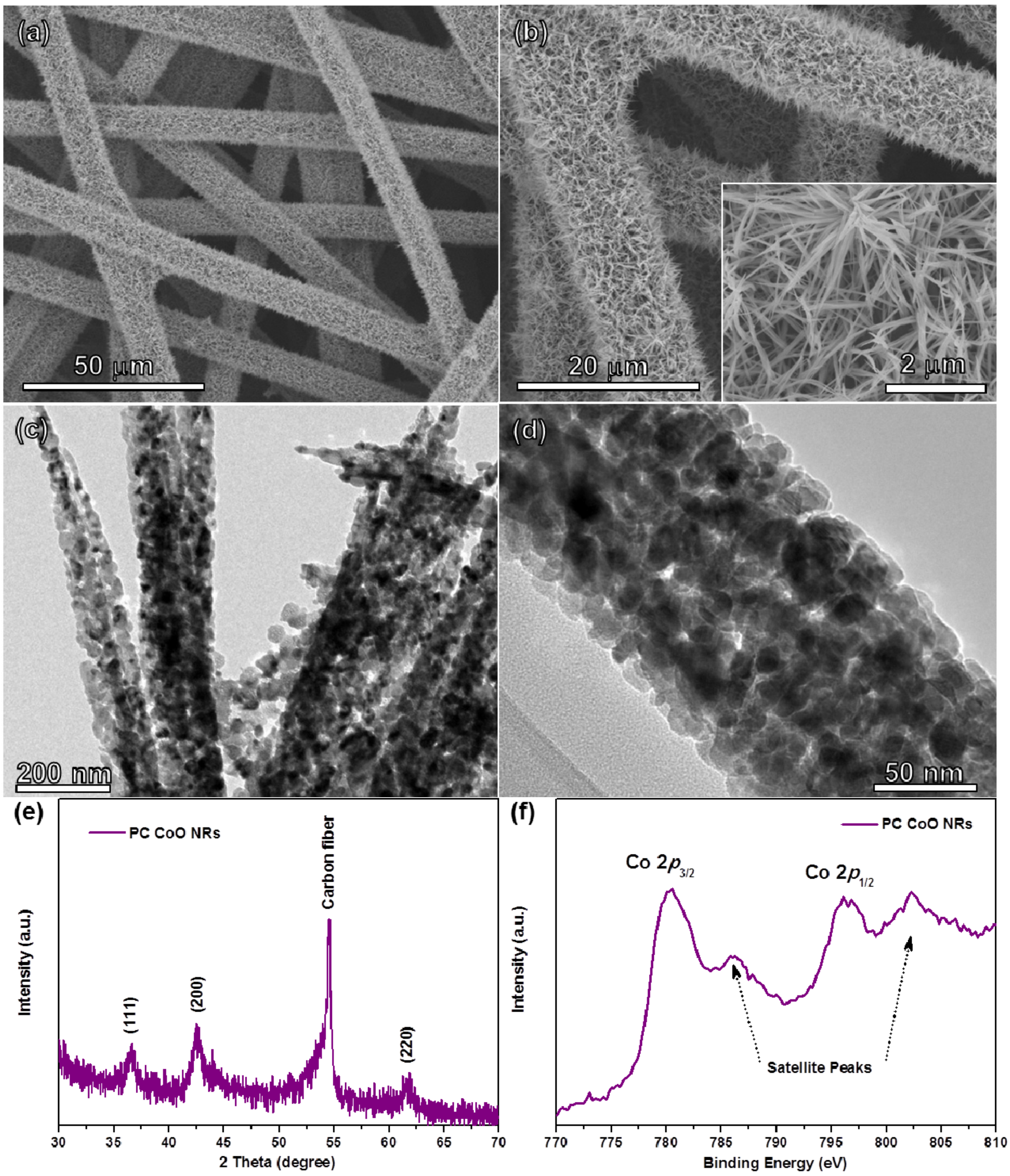

Supplementary Figure 10. Characterization of PC CoO NRs. (**a**) and (**b**) Top-view SEM images of PC CoO NRs on the CFP substrate synthesized under hydrothermal conditions. (**c**) Low-magnification and (**d**) high-magnification TEM images of PC CoO NRs, showing that PC CoO NRs consist of loose grains with sizes of 10-30 nm. (**e**) XRD and (**f**) Co 2*p* XPS spectra of PC CoO NRs. The satellite peaks indicated by the arrows in (**f**) confirm the presence of Co^{2+}.

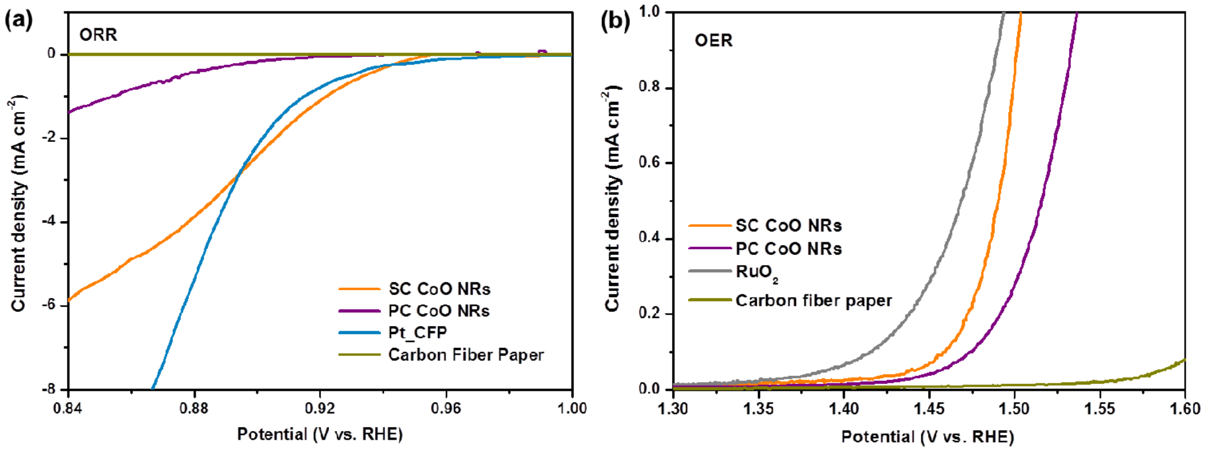

Supplementary Figure 11. (**a**) and (**b**) Linear sweep voltammetry (LSV) curves measured for ORR and OER on SC CoO NRs and control samples in O_2-saturated 1 M KOH solution at scan rate of 0.5 mV s^{-1}, respectively, showing the onset potentials in the ORR and OER regions.

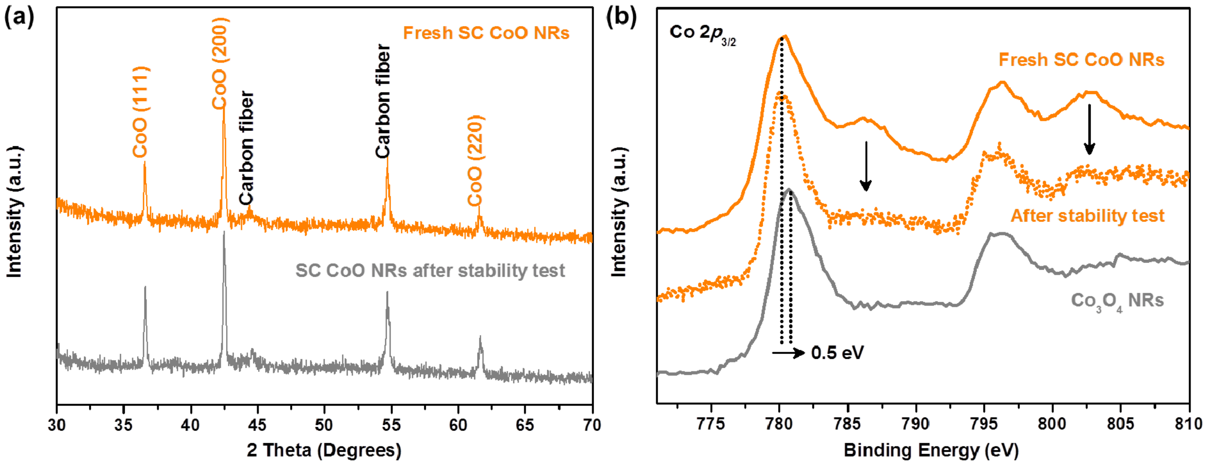

Supplementary Figure 12. Characterization of SC CoO NRs after long-term ORR stability test. (**a**) XRD and (**b**) XPS Co 2*p* spectra of SC CoO NRs before and after stability tests, and in (**b**) the fully oxidized PC CoO NRs (Co_3O_4 NRs) were employed as a reference. No obvious peaks attributed to Co_3O_4 phase was observed in (**a**), indicating that NRs after stability test are mainly CoO. Nevertheless, the decreased satellite peaks in (**b**) suggest a slight surface oxidation of CoO through ORR catalysis in KOH, which was also observed in previous work[4].

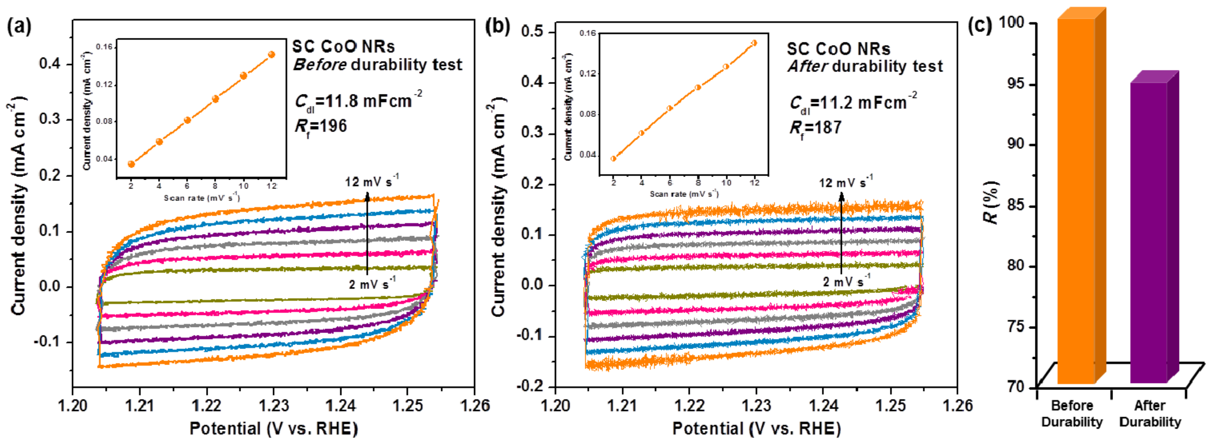

Supplementary Figure 13. Determination of active surface area of SC CoO NRs (**a**) before and (**b**) after durability test. The insets in (**a**) and (**b**) are plots of the current density at 1.23 V (with respect to the reversible hydrogen electrode, RHE) versus the scan rate to determine the double-layer capacitance (C_{dl}). The rough factor (R_f) values were determined by dividing C_{dl} by the value of 60 μF cm^{-2} (see detailed calculation in supplementary Methods). (**c**) The ratio (R) of active surface area before and after durability test.

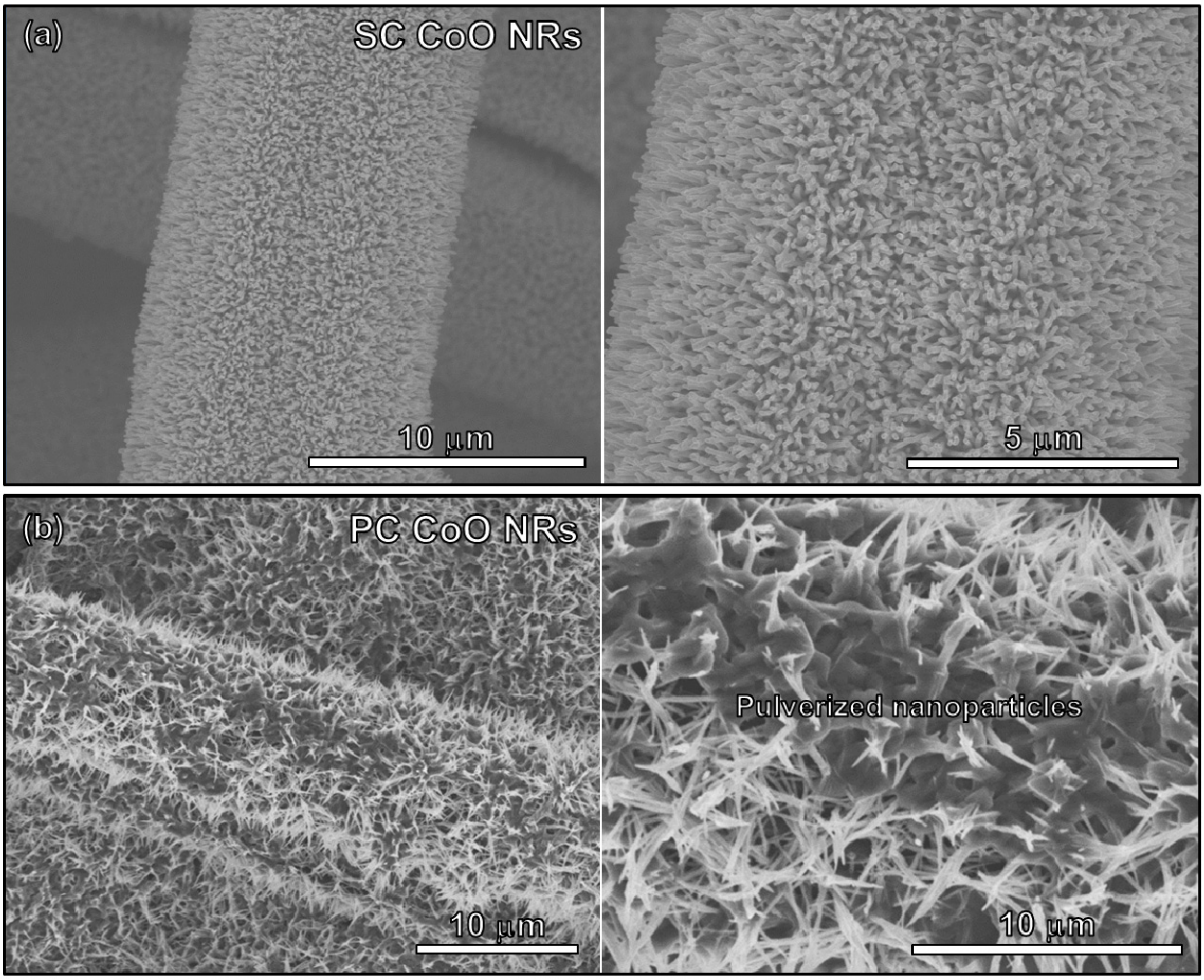

Supplementary Figure 14. (**a**) and (**b**) SEM images of SC and PC CoO NRs after 3000 cyclic voltammetry (CV) catalytic tests with accelerated scan rate of 100 mV s^{-1} in ORR region (0.5-1.1 V_{RHE}). As can be seen, after a test involving 3000 cycles, SC CoO NRs still retain the original one-dimensional morphology, however PC CoO NRs begin to collapse, indicating their lower electrochemical stability. Note that prior SEM analysis the samples of SC and PC CoO NRs were well dried in vacuum.

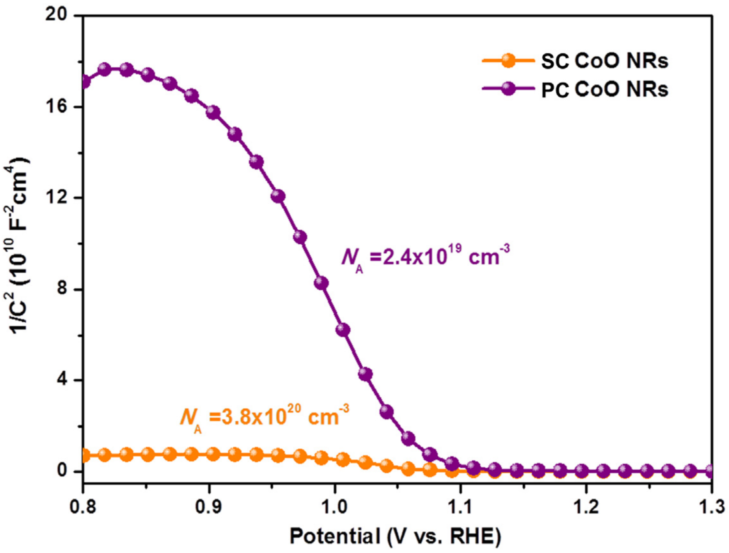

Supplementary Figure 15. Mott-Schottky (M-S) plots obtained for SC and PC CoO NRs. The detailed analysis of the M-S plots is provided in Supplementary Note 3.

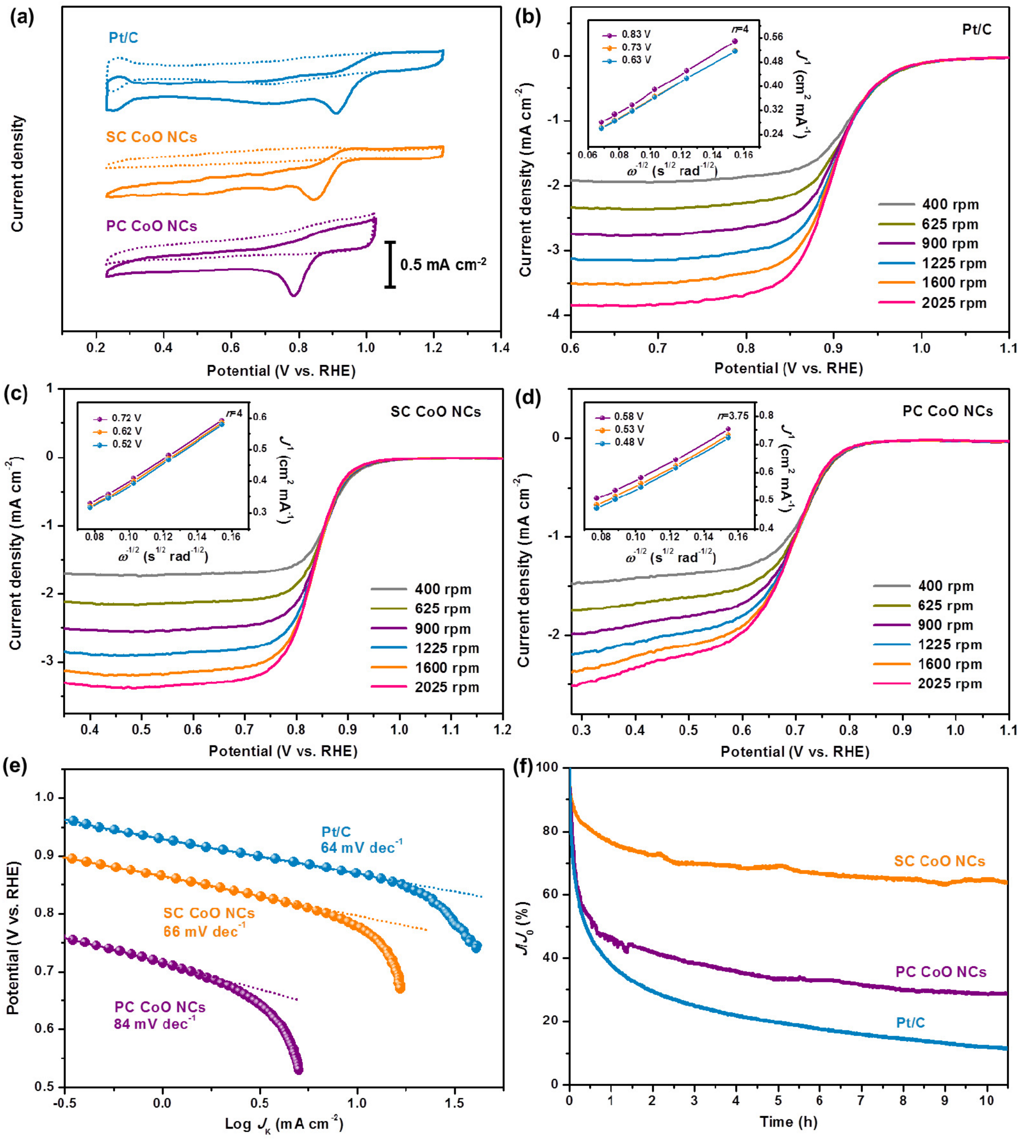

Supplementary Figure 16. The ORR catalytic activity of SC CoO NCs coated on a glass carbon electrode. (**a**) CV curves measured for SC and PC CoO NCs and for commercial 20 % Pt/C in O_2-saturated or Ar-saturated 1 M KOH. Rotating disk electrode (RDE) voltammograms recorded for (**b**) Pt/C, (**c**) SC and (**d**) PC CoO NCs in O_2-saturated 1 M KOH at the different rotation rates, and the insets in (**b**)-(**d**) show the corresponding Koutecky-Levich (K-L) plots (J^{-1} *vs.* $\omega^{-1/2}$) at different potentials. (**e**) The corresponding Tafel plots for SC and PC CoO NCs and for Pt/C obtained from the corresponding RDE data. (**f**) Chronoamperometric (CA) response of SC, PC CoO NCs and Pt/C catalysts at a constant voltage of 0.60 V_{RHE}. The scan rate for CV and RDE was 5 mV s^{-1}. Catalyst loading was 0.40 mg cm^{-2} for all samples.

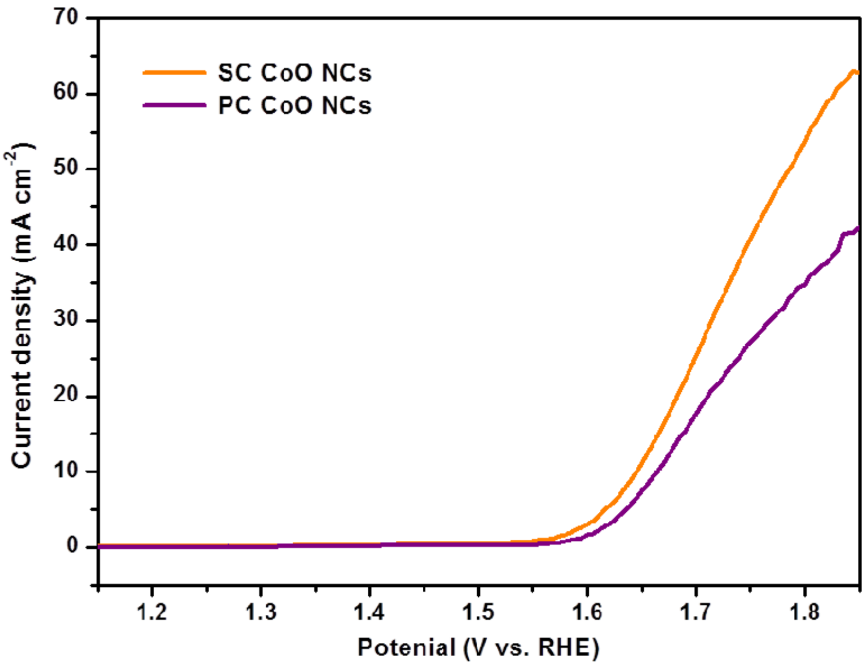

Supplementary Figure 17. The OER LSVs measured for SC and PC CoO NCs coated on a glass carbon electrode in O_2-saturated 1 M KOH with the same mass loading of 0.4 mg cm^{-2}.

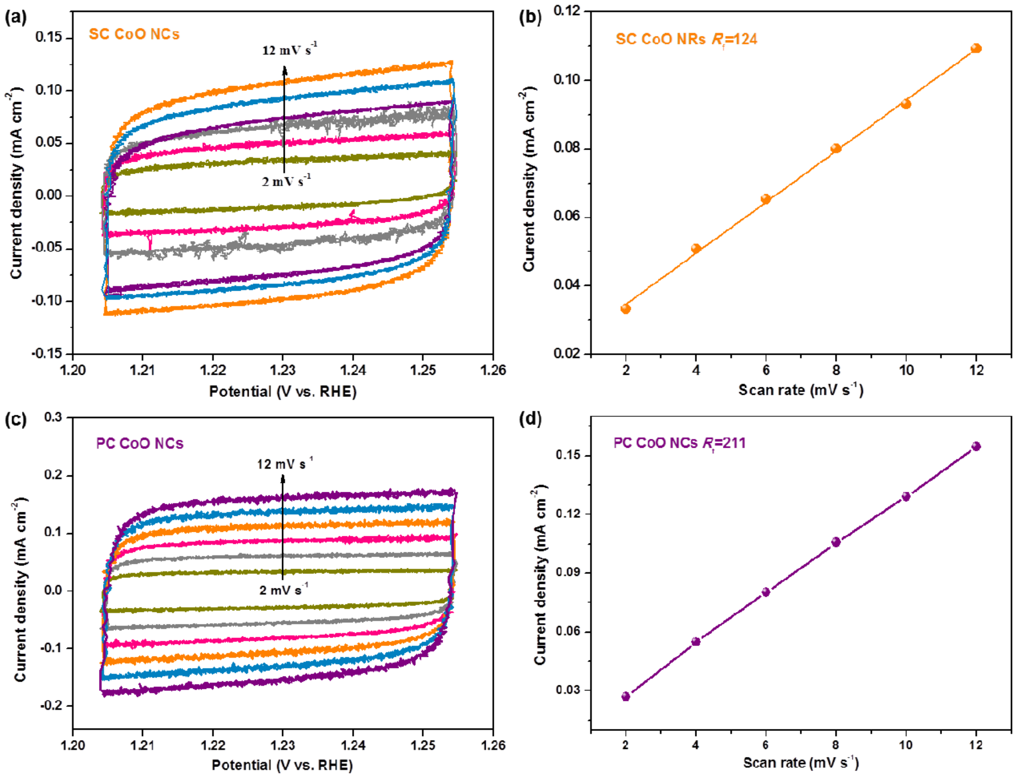

Supplementary Figure 18. (**a**) and (**c**) CVs measured for SC and PC CoO NCs coated on RDE in 1 M KOH at scan rates of 2 to 12 mV s^{-1}, respectively. (**b**) and (**d**) Plot of the current density at 1.23 V_{RHE} versus the scan rate to determine R_f.

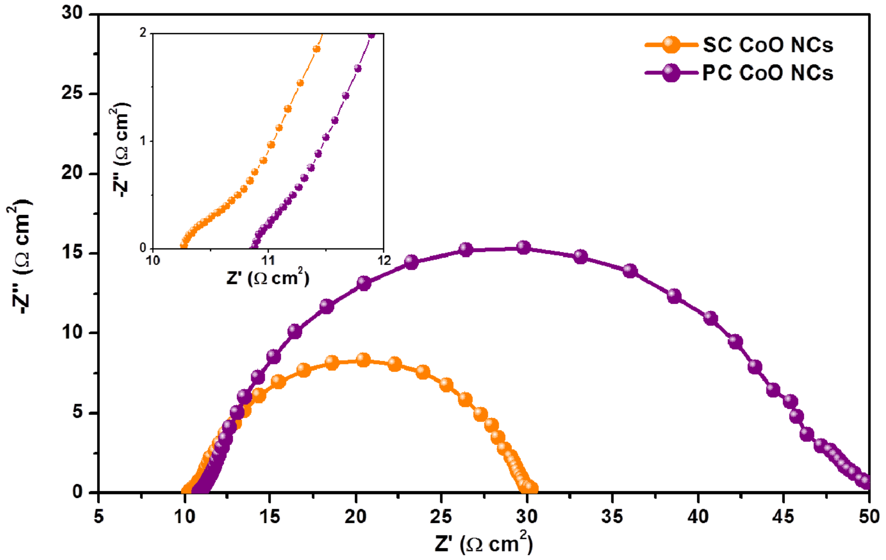

Supplementary Figure 19. Electrochemical impedance spectroscopy (EIS) data obtained for SC and PC CoO NCs at 0.8 V_{RHE}. The SC CoO NC materials showed a smaller semicircle than that measured for PC CoO NCs, suggesting a smaller charge transfer resistance in SC CoO NCs during catalytic reactions due to higher electronic conductivity of SC NCs in comparison to that of PC NCs.

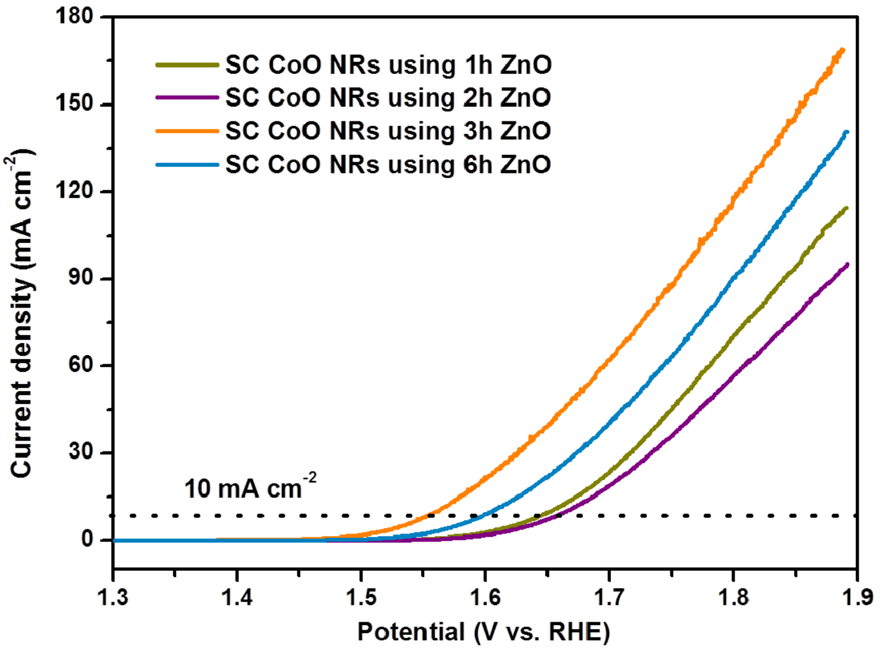

Supplementary Figure 20. The polarization curves obtained for SC CoO NRs prepared by using ZnO NRs grown at different periods of time. Obviously, the 3 h sample exhibits the lowest onset potential and largest OER current. A better performance of the 3 h sample in comparison to the 6 h sample (with nearly the same NR length) is mainly due to the presence of larger amount of holes in NRs of the 3 h sample (Supplementary Fig. 5).

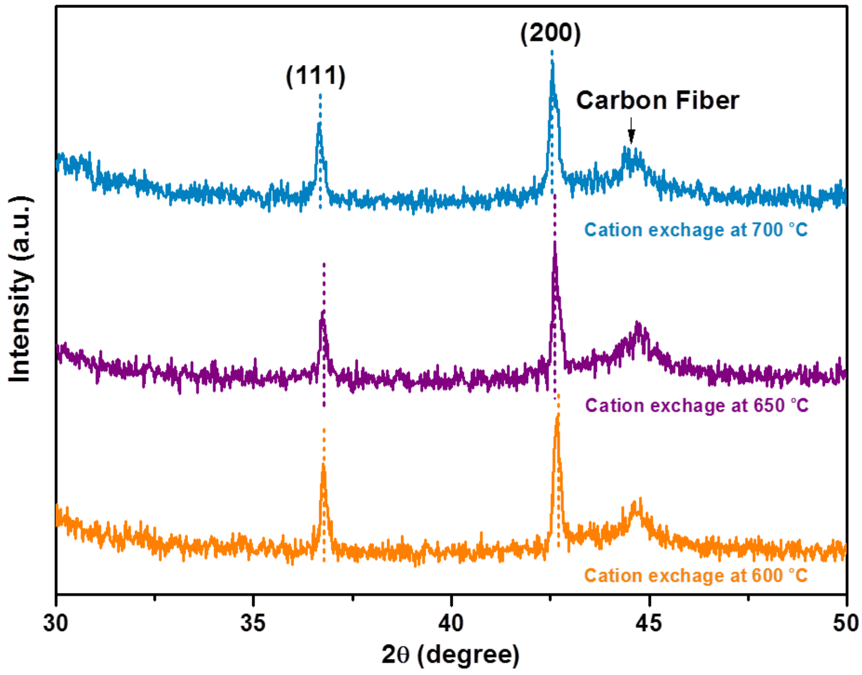

Supplementary Figure 21. XRD spectra of SC CoO NRs obtained via cation exchange at different temperatures.

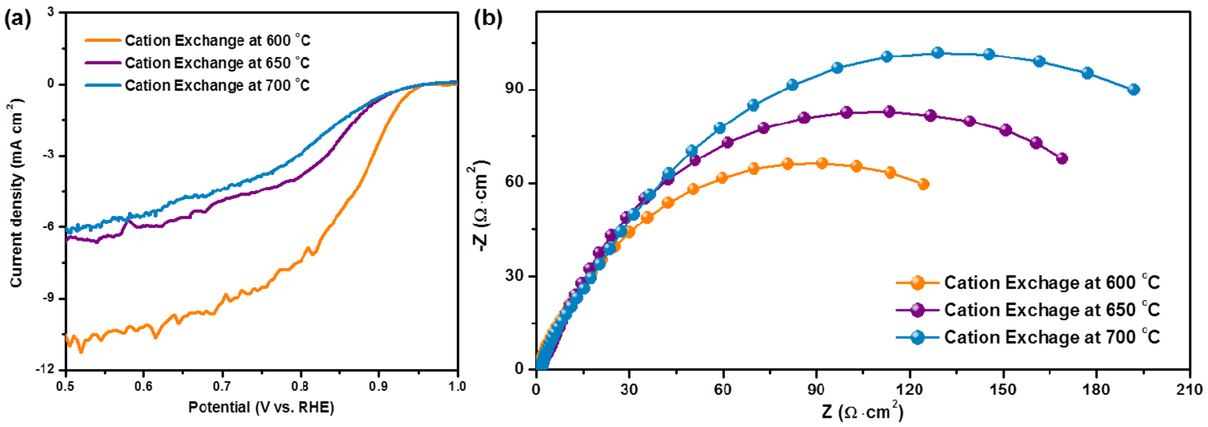

Supplementary Figure 22. (**a**) ORR polarization curves and (**b**) EIS spectra (measured at 0.7 V_{RHE}) of SC CoO NRs obtained via cation exchange at different temperatures.

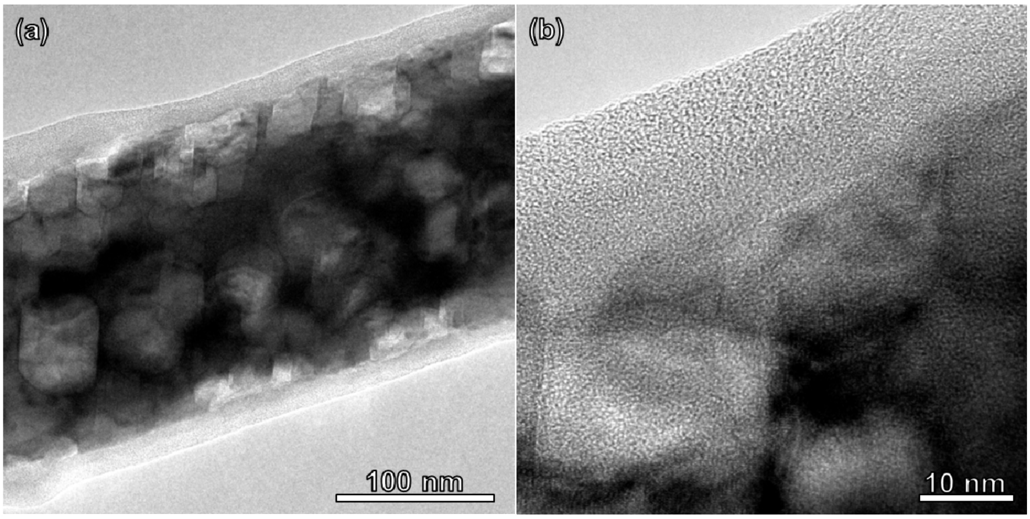

Supplementary Figure 23. (**a**) and (**b**) Typical low magnification TEM and HRTEM images of CoO NRs obtained via cation exchange at higher temperature, respectively.

Supplementary Tables

Supplementary Table 1. The Brunauer-Emmett-Teller (BET) surface area, best fit values of XPS O 1*s* peak for SC and PC CoO NRs, and atomic ratios of Co:O, Co:O_C and Co:O_L. The XPS O1*s* spectra are shown in Supplementary Fig. 7.

Sample	BET Surface Area (m^2 g^{-1})	Lattice Oxygen (O_L)	Chemisorbed Oxygen (O_C)		Atomic Ratio Co:O	Atomic Ratio Co:O_C	Atomic Ratio Co:O_L
		Peak I	Peak II	Peak III			
		RA (%)	RA (%)	RA (%)			
**SC CoO NRs**	**27**	**32.3**	**52.3**	**15.4**	**1:3.24**	**1:2.19**	**1:1.05**
**PC CoO NRs**	**85**	**54.7**	**35.4**	**9.9**	**1:1.81**	**1:0.82**	**1:0.99**

RA: relative area of the peak.

Supplementary Table 2. Summary of electro-catalytic activity of the recently reported highly active ORR/OER bi-functional oxygen electrode materials.

Catalyst	OER Onset Potential (V_{RHE})	OER Tafel Slope (mV dec^{-1})	OER Potential @ 10 mA cm^{-2} ($E_{J=10}$) (V_{RHE})	ORR Onset Potential (V_{RHE})	ORR Tafel Slope (mV dec^{-1})	Overall Oxygen Electrode Activity (V) ($\Delta E = E_{j=10} - E_{1/2}$)	Catalyst Loading (mg cm^{-2})	Electrolyte	Substrate	Ref
**SC CoO NRs**	**1.47**	**44**	**1.56**	**0.96**	**47**	**0.71**	**0.4**	**1 M KOH**	**CFP**	**This work**
CoO/N-doped grapheme oxide	1.30	71	1.57	0.90	48	0.76	0.7	1 M KOH	Glassy carbon	[5]
Co_3O_4/N-doped graphene	1.40	67	1.54	0.86	37	0.71	1.0	1 M KOH	Ni foam	[6]
Co_3O_4/porous carbon	1.47	70	1.52	0.98	89	0.74	0.2	0.1 M KOH	Cu foil	[7]
Mn_xO_y/N-doped carbon	1.55	82.6	1.68	0.85	N.A.	0.87	0.21	0.1 M KOH	Glassy carbon	[8]
Cu-MOF/graphene oxide	1.19	65	1.59	0.29	69	N.A.	0.23	0.5 M H_2SO_4	Glassy carbon	[9]
H-Pt/$CaMnO_3$	1.50	N.A.	1.80	0.90	65	1.01	0.1	0.1 M KOH	Glassy carbon	[10]
$CaMn_4O_x$	1.30	N.A.	1.77	0.85	N.A.	1.04	N.A.	0.1 M KOH	Glassy carbon	[11]
O-NiCoFe-LDH	1.49	93	1.57	0.80	60	0.94	0.12	0.1 M KOH	Glassy carbon	[12]
PCN	1.53	61.6	1.63	0.94	122.3	0.96	0.2	0.1 M KOH	CFP	[13]
N-doped graphene/CNT	1.50	83	1.63	0.88	N.A.	1.00	0.25	0.1 M KOH	Glassy carbon	[14]

Supplementary Table 3. Electrochemical analysis of SC and PC CoO NCs and their intrinsic activities toward ORR and OER.

Sample	Double-layer Capacitance (C_{dl}) (mF cm^{-2})	Rough Factor (R_f)	Electrochemically Active Surface Area[a] (ECSA) (m^2 g^{-1})	ORR		OER	
				J_k @ 0.6 V_{RHE} (mA cm^{-2})	$J_{k, specific}$[b] @ 0.6 V_{RHE} (mA cm^{-2})	J @ 1.65 V_{RHE} (mA cm^{-2})	$J_{specific}$[c] @ 1.65 V_{RHE} (mA cm^{-2})
**SC CoO NCs**	**7.45**	**124**	**31**	**17.8**	**0.144**	**11.1**	**0.090**
**PC CoO NCs**	**12.66**	**211**	**53**	**4.2**	**0.020**	**7.3**	**0.035**

[a]ECSA was calculated using $\text{ECSA} = \frac{R_f}{m_{\text{loading}}}$, where m_{loading} is the loading mass of catalyst per geometrical area of the electrode.

[b]According to the reference[15], $J_{k,\ specific}$ was calculated using $J_{k,\text{specific}} = \frac{I_k}{S_{\text{catalyst}}} = \frac{I_k}{S_{\text{geo}} \cdot R_f} = \frac{J_k}{R_f}$, where I_k is the ORR kinetic current, $S_{catalyst}$ and S_{geo} are the real active surface area of catalyst and the geometrical area of the electrode, respectively.

[c]$J_{specific}$ was calculated using $J_{\text{specific}} = \frac{I}{S_{\text{catalyst}}} = \frac{I}{S_{\text{geo}} \cdot R_f} = \frac{J}{R_f}$, where I is the OER current.

Supplementary Table 4. Frequency analysis of the intermediates on {110} surface of CoO.

	**OOH***	**O***	**OH***
**Computed Vibrational Frequencies (meV)**	377.0617	75.27816	463.2084
	184.0485	48.19039	100.8558
	104.9017	24.28806	67.65042
	82.4917		50.37478
	54.05606		44.45995
	45.6425		19.00986
	31.97415		
	17.6241		
	13.26342		
***T*S* (eV)**	0.11	0.04	0.06
**E_{ZPE} (eV)**	0.46	0.07	0.37

Supplementary Table 5. Free energy of different facets of CoO with and without intermediates.

	State	**{100}**	**{110}**	**{111}-O**	**{111}-Ov**
**Free Energy (eV)**	**Clean Surface**	-747.36	-360.53	-853.16	-848.48
	**OOH***	-759.65	-374.67	-866.52	-863.39
	**O***	-752.11	-366.95	-857.04	-854.01
	**OH***	-760.32	-370.55	-861.44	-858.43

Supplementary Table 6. Summary of the recently reported highly active TMO-based ORR catalysts.

Catalyst	Onset Potential (V_{RHE})	Half-wave Potential (V_{RHE})	Tafel Slope (mV dec^{-1})	Catalyst Loading (mg cm^{-2})	Electrolyte	Reference
SC CoO NCs	0.92	0.83	66	0.4	1 M KOH	This work
Co_3O_4/N-doped graphene	0.88	0.83	42	0.17	0.1 M KOH	[6]
CoO NPs/ NCNT	0.93	0.85	-	0.1	1 M KOH	[4]
CoO/N-doped grapheme oxide	0.90	0.78	48	-	1 M KOH	[5]

Supplementary Table 7. O-vacancy concentration, δ, of SC CoO NRs exchanged at different temperatures.

Sample	Cation Exchange Temperature (^{o}C)	Lattice Constant[a] a (Å)	Composition	δ[b]
1	600	4.234	$CoO_{0.97}$	0.03
2	650	4.236	$CoO_{0.91}$	0.09
3	700	4.245	$CoO_{0.82}$	0.18

[a]From XRD measurement.

[b]Average data from inductively coupled plasma mass spectrometry (ICP-MS) measurements.

Supplementary Notes

Supplementary Note 1. Estimation of Surface Area Ratio of {111} Facets

As shown in Supplementary Fig. 6, the surface area of an individual nanopyramid, consisting of two {100} and two {111} facets, can be expressed as follows:

$$\begin{aligned} S_{\text{Pyramid}} &= 2S_{\{100\}} + 2S_{\{111\}} \\ &= 2\times a^2 + 2\times\frac{\sqrt{3}}{2}a^2 \\ &= (2+\sqrt{3})a^2 \end{aligned} \quad (1)$$

where a is the length of the square side shown in Supplementary Fig. 6. Therefore, the surface ratio of {111} facets is:

$$R_{\{111\}} = \frac{2S_{\{111\}}}{S_{Pyramid}} = \frac{\sqrt{3}a^2}{(2+\sqrt{3})a^2} \approx 46\% \quad (2)$$

Supplementary Note 2. XPS Analysis

The information provided by XPS for inorganic materials is below 2-4 nm from the outermost surface[16], which is about 17-33 alternate Co and O atomic planes for CoO from <111> direction. As can be seen in Supplementary Table 1, the atomic ratio of Co:O_L is 1:1.05, indicating a larger number of oxygen species exposed on the surface of SC CoO NRs. It is assumed that the exposed {111} nanofacets of SC CoO NRs are O-terminated, and the theoretical atomic ratio of Co:O_L for {111} facets of SC CoO NRs can be calculated as follows:

$$\frac{8}{9} \le \text{Atomic Ratio Co:O}_{\text{L}} = \frac{\text{Number of Co Atomic Planes}}{\text{Number of O Atomic Planes}} \le \frac{16}{17} \quad (3)$$

As estimated in Supplementary Note 1, {111} and {100} facets account for 46 % and 54 % of the surface area, respectively. Moreover, since {100} facets are not polar, the atomic ratio of Co:O_L should be 1:1. Hence, the overall atomic ratio of Co:O_L for SC CoO NRs is calculated as:

$$\frac{1}{1.06} = \frac{8\times54\%+8\times46\%}{8\times54\%+9\times46\%} \le \text{Atomic Ratio Co:O}_{\text{L}} \le \frac{16\times54\%+16\times46\%}{16\times54\%+17\times46\%} = \frac{1}{1.03} \quad (4)$$

The experimental atomic ratio of Co:O_L for SC CoO NRs is 1:1.05, which exactly falls into this range. Thus, the exposed {111} nanofacets of SC CoO NRs should be O-terminated.

Moreover, the much smaller Co:O_C ratio for SC CoO NRs as compared with that for PC CoO NRs (Supplementary Table 1) is ascribed to a large quantity of water molecules and oxygen species adsorbed onto the {111}-Ov facets of SC CoO NRs.

Supplementary Note 3. Mott-Schottky Analysis

The acceptor density can be calculated from the slopes of the M-S plots (Supplementary Fig. 15) by the following equation[17],

$$\frac{dC^{-2}}{dV} = \frac{-2}{q\varepsilon_0\varepsilon_r N_A A^2} \quad (5)$$

where A is the surface area of the sample studied, ε_r is the dielectric constant of CoO with the value of 5.4. The acceptor concentrations in SC and PC CoO NRs were estimated to be 3.8×10^{20} and 2.4×10^{19} cm^{-3}, respectively, indicating a greatly enhanced carrier concentration in SC CoO NRs provided by the single-crystalline nature and the O-vacancies present on the surface of SC CoO NRs.

Supplementary Note 4. Analysis of the Intrinsic ORR/OER Activity of SC CoO NCs

SC CoO NCs with sizes of about 50 nm were coated onto a RDE to test their intrinsic ORR/OER performances in comparison with those of PC CoO NCs, the state-of-art 20 % Pt/C and RuO_2 catalysts (Supplementary Fig. 16 and Supplementary Fig. 17).

The intrinsic ORR catalytic activity of SC CoO NCs was first evaluated by CV. As can be seen in Supplementary Fig. 16a, SC CoO NCs exhibit an ORR onset potential of 0.92 V_{RHE}. Although this value is not as good as than that of commercial 20 % Pt/C (~1 V_{RHE}), it is better than that of PC CoO NCs with larger ECSA (Supplementary Fig. 18 and Supplementary Table 3) and most of transition metal oxide catalysts (Supplementary Table 6). The RDE measurements were further carried out to reveal the ORR kinetics of SC CoO NCs (Supplementary Fig. 16c). The electron transfer number (n) was calculated to be 4 at 0.52-0.72 V_{RHE} from the slopes of Koutecky-Levich plots (Supplementary Fig. 16c, inset), suggesting SC CoO NCs favour 4e oxygen reduction process, similar to that observed on a commercial Pt/C catalyst (Supplementary Fig. 16b, inset). Moreover, as shown in Fig. 5a and 5b, and described in the main text, SC CoO NCs exhibit a kinetic current density (J_k) of 17.8 mA cm^{-2} and a specific kinetic current density ($J_{k,\ specific}$) of 0.144 mA cm^{-2} at 0.6 V_{RHE}, which are 4.2 and 7.2 times

larger than those obtained for PC CoO NCs, respectively. The excellent ORR activity of SC CoO NCs is further supported by the small Tafel slope of 66 mV decade^{-1} at low overpotentials, which is much smaller than that obtained for PC CoO NCs and approach the value determined for Pt/C (64 mV decade^{-1}). This fact is fairly consistent with the results obtained for catalysts supported on CFP (Fig. 4b). Besides, SC NCs also exhibit better stability than PC NCs and Pt/C (Supplementary Fig. 16f).

Moreover, the intrinsic OER catalytic activity of SC CoO NCs was also tested in comparison to PC CoO NCs. As shown in Supplementary Fig. 17, SC CoO NCs afford lower onset potential and higher OER oxidation current than the corresponding values obtained for PC CoO NCs. Moreover, as shown in Fig. 5c and 5d, and described in the main text, the current density (J) and specific current density ($J_{specific}$) at 1.65 V_{RHE} of SC CoO NRs are about 1.5 and 2.6 times larger than those of PC CoO NCs, respectively.

These results unambiguously demonstrate that the specific pyramidal structure, and highly exposed vacancy-rich {111} facets play the key role in enhancing the intrinsic ORR/OER activity of SC CoO NCs.

Supplementary Note 5. Tuning the Concentration of O-vacancies in SC CoO NCs

The concentration of O-vacancies in SC CoO NRs can be controlled through tuning the cation exchange temperature (Supplementary Fig. 21). The structural and compositional data for SC CoO NRs obtained via cation exchange at different temperatures are summarized in supplementary Table 7. As can be seen from this table, higher exchange temperature favors the formation of O-vacancies. Notably, the exchange temperatures lower than 600 ^{o}C cannot assure a complete conversion of ZnO into CoO. The ORR activity measurements show that SC CoO NRs obtained via cation exchange at 600 ^{o}C outperformed the samples prepared at 650 and 700 ^{o}C having higher concentration of O-vacancies (Supplementary Fig. 22a). HRTEM (Supplementary Fig. 23) and EIS (Supplementary Fig. 22b) characterizations reveal that the high cation exchange temperature damages the surface and decreases the electronic conductivity of SC CoO NRs. Our results are in good agreement with previous reports showing that the presence of excessive oxygen defects can inevitably cause the structural instability and decrease the electronic conductivity of metal oxides[18-20]. These collective results demonstrate that the most efficient CoO NRs are obtained via cation exchange at 600 ^{o}C and feature a modest concentration of O-vacancies.

Supplementary Methods

Experiment Section

Synthesis of ZnO NRs on CFP substrate. Prior to the synthesis, a hydrophilic commercial CFP was cleaned by rinsing with water and ethanol and dried. Then, ZnO NRs were grown on CFP under hydrothermal conditions[21]. The length of ZnO NRs was simply tuned by controlling the growth time. Specifically, growth for 1 h, 2 h and 3 h yielded the lengths of NRs of 50 nm, 500 nm and 1.6 μm, respectively (Supplementary Fig. 4), and further prolongation of the growth time did not produce longer NRs. An optimum growth time of 3 h was determined and used in this study (Supplementary Fig. 20).

Synthesis of PC CoO NRs on CFP substrate. PC CoO NRs were grown on CFP under hydrothermal conditions[22]. Firstly, a seed layer was deposited on the CFP substrate by the dip-coating method using 0.4 M $CoCl_2$ and 2 M $CO(NH_2)_2$ in ethanol. The seeded CFP substrate was then calcined at 450 °C for 4 h. Secondly, the precursor NR arrays were grown in the aqueous solution of 0.04 M $CoCl_2$ and 0.2 M $CO(NH_2)_2$ at 90 °C for 6 h. Finally, the as-grown products were annealed under a constant flow of Ar gas (50 sccm) at 425 °C for 3 h and 600 °C for 1 h to obtain pure CoO NRs. The loading mass of PC CoO NRs on CFP was controlled by changing the thickness of the seed layer and the growth time of NR arrays. The loading mass of the sample in Fig. 4 was ~0.21 mg cm^{-2}.

Synthesis of Pt catalysts on CFP substrate. Pt catalysts onto the CFP substrate were obtained by magnetron sputtering followed by annealing in Ar gas to enhance adhesion between Pt layer and the CFP substrate. The Pt loading was controlled by the deposition rate and time. The loading mass of the sample in Fig. 4 was ~0.18 mg cm^{-2}.

Loading of commercial RuO_2 on CFP substrate. 5 mg of RuO_2 (Alfa Aesar) and 40 μL of 5 wt% Nafion solution were dispersed in 1 mL of ethanol by at least 30 min sonication to form a homogeneous ink. About 0.2 mg of RuO_2 was loaded per 1 cm^2 of CFP.

Synthesis of SC CoO NCs. SC CoO NCs with sizes of ~50 nm were fabricated on a fluorine-doped tin oxide (FTO) substrate using the same method as preparations performed on CFP, and then carefully scaped from FTO.

Synthesis of PC CoO NCs. PC CoO NCs were grown in the aqueous solution of 0.04 M $CoCl_2$ and 0.2 M $CO(NH_2)_2$ at 90 °C for 2 h. Afterwards, the as-grown products were annealed under a constant flow of Ar gas (50 sccm) at 425 °C for 3 h and 600 °C for 1 h to obtain PC CoO NCs.

Characterization Section

Structural characterization. XPS analysis was performed using a PHI Quantum 2000 scanning ESCA Microprobe spectrometer. XRD characterization was carried out using a Bruker D8 advance XRD. The mass loading of as-obtained catalysts on CFP was determined using inductively coupled plasma mass spectrometry (ICP-MS, Perkin-Elmer, NexION 300Q). BET surface area was determined from nitrogen adsorption data measured at 77 K on an ASAP 2020 physisorption analyser (Micromeritics, Inc., USA).

Electrochemical characterization the ORR/OER performance of catalysts supported on CFP. The electrochemical measurements were performed in a three-electrode electrochemical cell using an Hg/HgO electrode in saturated KCl solution as the reference electrode, Pt plate as the counter electrode and the CFP electrode as the working electrode. A flow of O_2 was maintained over the electrolyte (1.0 M KOH) during measurements in order to ensure the O_2/H_2O equilibrium at 1.23 V_{RHE}. The potential was calibrated with respect to RHE in the high purity hydrogen saturated electrolyte with a Pt wire as the working electrode. M-S plots were obtained using a Versastat 3 potentiostats electrochemistry workstation with a frequency of 1000 Hz.

Electrochemical characterization of the intrinsic activities of catalysts toward ORR/OER. To investigate the intrinsic ORR/OER activities of catalysts, 5 mg of a catalyst, 2 mg of a carbon powder (Vulcan XC 72) and 40 μL of 5 wt% Nafion solution were dispersed in 1 mL of water by sonication for at least 30 min to form a homogeneous ink. Then, the catalyst ink was loaded onto a glassy carbon electrode to achieve a mass loading of about 0.4 mg cm^{-2}. CV, LSV, RDE and CA measurements were carried out using a Pine potentiostat in a three-electrode electrochemical cell using an Hg/HgO electrode in saturated KCl solution as the reference electrode, and Pt plate as the counter electrode. At the start of each measurement, 1 M KOH electrolyte was bubbled with O_2 for 30 min to form an O_2-saturated environment, and the entire measurement was carried out under continuous flow of O_2. Before the CV, LSV, and RDE tests, the working electrode was firstly cycled several times to achieve a stable response, and the further measurements were carried out at the scan rate of 5 mV s^{-1}. The values of C_{dl} were determined from the slope of the

capacitive current versus the scan rate and divided by the value of 60 μF cm^{-2} to obtain R_f[23]. EIS measurements were conducted on a Versastat 3 electrochemical workstation by applying an AC voltage with 5 mV amplitude within the frequency range of 0.01 to 200 kHz.

In ORR process, the electron transfer number, *n*, per O_2 molecule can be calculated from the slope of Koutecky-Levich plot using the following equation[6],

$$\frac{1}{J}=\frac{1}{J_k}+\frac{1}{B\omega^{1/2}} \qquad (6)$$

where J is the measured current density on RDE, J_k is the kinetic current density at a constant potential, ω is the electrode rotating speed, and B is the reciprocal of the slope. B can be determined from the slope of Koutecky-Levich plot using the following expression:

$$B = 0.62nF\nu^{-1/6}C_{O_2}D_{O2}{}^{2/3} \qquad (7)$$

where n is the number of electrons transferred per oxygen molecule, F is the Faraday constant, ν is the kinetic viscosity, C_{O_2} is the bulk concentration of O_2, and D_{O2} is the diffusion coefficient of O_2 in 1 M KOH.

Computation Section

Introduction. All density functional theory (DFT) calculations were performed using the Vienna Ab-initio Simulation Package (VASP) package[24,25]. An effective U parameter of 3.7 eV was applied for Co 3*d* states under the approximation introduced by Dudarev *et al.*[26] to describe well the electronic structure of CoO. The projector augmented wave (PAW)[27,28] pseudopotential and the Perdew-Burke-Ernzerhof (PBE) exchange-correlation functional[29] were used in the calculations with a 400 eV plane-wave cut-off energy. All structures in the calculations were spin-polarized and relaxed until the convergence tolerance of force on each atom was smaller than 0.05 eV. The energy converge criteria was set to be 10^{-5} eV for self-consistent calculations with a gamma-centre 2x2x1 *k*-mesh. All periodic slabs have a vacuum spacing of at least 15 Å. The structural model of CoO {100} facet contains four Co-O layers (128 atoms), with a supercell size of $a=b=12.07$Å, $c=21.80$Å, $\alpha=\beta=\gamma=90^{o}$, while {110} facet consists of four Co-O layers (64 atoms), with $a=8.53$Å, $b=12.07$ Å, $c=20.05$Å, $\alpha=\beta=\gamma=90^{o}$. The {111}-O surface model is composed of four Co layers and five O layers (160 atoms), with $a=b=12.07$Å, $c=29.78$Å, $\alpha=\beta=90^{o}$, $\gamma=120^{o}$, and the {111}-Ov surface is obtained by removing one surface O atom (159

atoms). For {111}-O and {111}-Ov surfaces, one H layer was introduced to make the slab to obey the electron counting rule, which is a common method used for polar surfaces[30,31]. The top-view and side-view of the models are shown in Fig 6a. In calculations, the two bottom layers (plus the H layer for {111}-O and {111}-Ov) were kept fixed, whereas the rest of atoms were allowed to relax.

Calculation of O-vacancy formation energy. Three low-index surfaces, including {001}, {110} and {111}-O, were considered in the calculations. The O-vacancy formation energy E_{O_V} was calculated using the following definition[32]:

$$E_{O_V} = E_{Surf\text{-}O_V} + \frac{1}{2}E_{O_2} \text{-} E_{Surf} \quad (8)$$

where $E_{Surf\text{-}O_V}$ is the energy of a surface with an oxygen vacancy, E_{Surf} is the energy of a surface without oxygen vacancies and E_{O_2} is the energy of a gas phase O_2 molecule.

Free energy calculation. Both ORR and OER are assumed to involve four elementary reaction steps[33,34], and OER can be considered as a reverse process of ORR. In ORR, the reduction of O_2 to OH^- in alkaline media proceeds through following four reactions[33].

$$O_2(g) + 2H_2O + 4e^- + * \rightarrow OOH* + OH^- + H_2O(l) + 3e^- \quad (9)$$

$$OOH* + OH^- + H_2O(l) + 3e^- \rightarrow O* + 2OH^- + H_2O(l) + 2e^- \quad (10)$$

$$O* + 2OH^- + H_2O(l) + 2e^- \rightarrow OH* + 3OH^- + e^- \quad (11)$$

$$OH* + 3OH^- + e^- \rightarrow 4OH^- \quad (12)$$

where * refers to a given adsorption site in the specific facet. The free energy of ORR can be computed by the following equation:

$$\Delta G = \Delta E + \Delta ZPE - T\Delta S \quad (13)$$

The value of ΔE was obtained by computation performed for specified geometrical structures, and the values of ΔZPE and ΔS were determined by using the computed vibrational frequencies for {110} surface of CoO (Supplementary Table 4), and the reactants and products in the gas phase[34]. Moreover, an external bias U was imposed on each step by including a -eU term in the computation of the reaction free energy. It is noteworthy that the exploration of active sites on

the specific facets was conducted and chosen according to the most energetically stable adsorption site. The calculated free energies of the adsorbed atoms/molecules on the active sites on different facets of CoO are listed in Supplementary Table 5.

Supplementary References

1. McIntyre, N. S. & Cook, M. G. X-ray photoelectron studies on some oxides and hydroxides of cobalt, nickel, and copper. *Analyt. Chem.* **47**, 2208-2213 (1975).
2. Jimenez, V. M., Fernandez, A., Espinos, J. P. & Gonzalezelipe, A. R. The state of the oxygen at the surface of polycrystalline cobalt oxide. *J. Electron Spect. Rel. Phen.* **71**, 61-71 (1995).
3. Bao, J. *et al.* Ultrathin spinel-structured nanosheets rich in oxygen deficiencies for enhanced electrocatalytic water oxidation. *Angew. Chem. Int. Ed.* **54**, 7399-7404 (2015).
4. Liang, Y. *et al.* Oxygen reduction electrocatalyst based on strongly coupled cobalt oxide nanocrystals and carbon nanotubes. *J. Am. Chem. Soc.* **134**, 15849-15857 (2012).
5. Mao, S. *et al.* High-performance bi-functional electrocatalysts of 3D crumpled graphene-cobalt oxide nanohybrids for oxygen reduction and evolution reactions. *Energy Environ. Sci.* **7**, 609-616 (2014).
6. Liang, Y. *et al.* Co_3O_4 nanocrystals on graphene as a synergistic catalyst for oxygen reduction reaction. *Nat. Mater.* **10**, 780-786 (2011).
7. Ma, T. Y., Dai S., Jaroniec, M. & Qiao, S. Z. Metal-organic framework derived hybrid Co_3O_4-carbon porous nanowire arrays as reversible oxygen evolution electrodes. *J. Am. Chem. Soc.* **136**, 13925-13931 (2014).
8. Masa, J. *et al.* Mn_xO_y/NC and Co_xO_y/NC nanoparticles embedded in a nitrogen-doped carbon matrix for high-performance bifunctional oxygen electrodes. *Angew. Chem. Int. Ed.* **53**, 8508-8512 (2014).
9. Jahan, M., Liu, Z. & Loh, K. P. A graphene oxide and copper-centered metal organic framework composite as a tri-functional catalyst for HER, OER, and ORR. *Adv. Func. Mater.* **23**, 5363-5372 (2013).
10. Han, X. *et al.* Hydrogenated uniform Pt clusters supported on porous $CaMnO_3$ as a bifunctional electrocatalyst for enhanced oxygen reduction and evolution. *Adv. Mater.* **26**, 2047-2051 (2014).
11. Gorlin, Y. & Jaramillo, T. F. A bifunctional nonprecious metal catalyst for oxygen reduction and water oxidation. *J. Am. Chem. Soc.* **132**, 13612-13614 (2010).
12. Qian, L. *et al.* Trinary layered double hydroxides as high-performance bifunctional materials for oxygen electrocatalysis. *Adv. Energy Mater.* **5**, 1500245 (2015).

13. Ma, T. Y. *et al.* Phosphorus-doped graphitic carbon nitrides grown in situ on carbon-fiber paper: flexible and reversible oxygen electrodes. *Angew. Chem. Int. Ed.* **54**, 4646-4650 (2015).
14. Tian, G.-L. *et al.* Nitrogen-doped graphene/carbon nanotube hybrids: in situ formation on bifunctional catalysts and their superior electrocatalytic activity for oxygen evolution/reduction reaction. *Small* **10**, 2251-2259 (2014).
15. Mayrhofer, K. J. J. *et al.* Measurement of oxygen reduction activities via the rotating disc electrode method: From Pt model surfaces to carbon-supported high surface area catalysts. *Electrochim. Acta* 53, 3181-3188 (2008).
16. Hüfner, S. (1995). Photoelectron spectroscopy: principles and applications. Springer Verlag.
17. Gleria, M. & Memming, R. J. Charge transfer processes at large band gap semiconductor electrodes: reactions at SiC-electrodes. *Electroanal. Chem. Interfacial* **65,** 163-175 (1975).
18. Cheng, F. *et al.* Enhancing electrocatalytic oxygen reduction on MnO_2 with vacancies. *Angew. Chem. Int. Ed.* **52**, 2474-2477 (2013).
19. Guo, Y. *et al.* Engineering the electronic state of a perovskite electrocatalyst for synergistically enhanced oxygen evolution reaction. *Adv. Mater.* **27**, 5989-5994 (2015).
20. Du, J. *et al.* Nonstoichiometric perovskite $CaMnO_{3-\delta}$ for oxygen electrocatalysis with high activity. *Inorg. Chem.* **53**, 9106-9114 (2014).
21. Law, M. *et al.* Nanowire dye-sensitized solar cells. *Nat. Mater.* **4**, 455-459 (2005).
22. Zhang, H., Ling, T. & Du, X.-W. Gas-phase cation exchange toward porous single-crystal CoO nanorods for catalytic hydrogen production. *Chem. Mater.* **27**, 352-357 (2015).
23. Nikolov, I. *et al.* Electrocatalytic activity of spinel related cobaltites $M_xCo_{3-x}O_4$ (M=Li, Ni, Cu) in the oxygen evolution reaction. *J. Electroanal. Chem.* **429**, 157-168 (1997).
24. Kresse, G. & Hafner, J. abinitio molecular-dynamics for liquid-metals. *Phys. Rev. B* **47**, 558-561 (1993).
25. Kresse, G. & Furthmuller, J. Efficiency of ab-initio total energy calculations for metals and semiconductors using a plane-wave basis set. *Comput. Mater. Sci.* **6**, 15-50 (1996).
26. Dudarev, S. L. *et al.* Electron-energy-loss spectra and the structural stability of nickel oxide: An LSDA+U study. *Phys. Rev. B* **57**, 1505-1509 (1998).

27. Blochl, P. E. Projector augmented-wave method. *Phys. Rev. B* **50**, 17953-17979 (1994).
28. Kresse, G. & Joubert, D. From ultrasoft pseudopotentials to the projector augmented-wave method. *Phys. Rev. B* **59**, 1758-1775 (1999).
29. Perdew, J. P., Burke, K. & Ernzerhof, M. Generalized gradient approximation made simple. *Phys. Rev. Lett.* **77**, 3865-3868 (1996).
30. Li, F., Feng, Y., Hu, Z., Xu J. & Zhang, L. Substrate engineering in stabilizing epitaxial MgO (1 1 1) polar ultrathin films: first-principles calculations. *J. Phys.: Condens. Matter* **26**, 315014 (2014).
31. Wang, Z., Pan, N., Li Z. & Yang, J. A first-principles study of ZnO polar surface growth: Adsorption of Zn_xO_y clusters. *J. Chem. Phys.* **139**, 124704 (2013).
32. Kushima, A., Yip, S. & Yildiz, B. Competing strain effects in reactivity of $LaCoO_3$ with oxygen. *Phys. Rev. B* **82**, (2010).
33. Jiao, Y., Zheng, Y., Jaroniec, M. & Qiao, S. Z. Origin of the electrocatalytic oxygen reduction activity of graphene-based catalysts: a roadnnap to achieve the best performance. *J. Am. Chem. Soc.* **136**, 4394-4403 (2014).
34. Norskov, J. K. *et al.* Origin of the overpotential for oxygen reduction at a fuel-cell cathode. *J. Phys. Chem. B* **108**, 17886-17892 (2004).
